# Force‐Induced Synergetic Pigmentary and Structural Color Change of Liquid Crystalline Elastomer with Nanoparticle‐Enhanced Mechanosensitivity

**DOI:** 10.1002/advs.202205325

**Published:** 2022-10-30

**Authors:** Chang Sun, Shuoning Zhang, YunXiao Ren, Jianying Zhang, Jiyuan Shen, Shengyu Qin, Wei Hu, Siquan Zhu, Huai Yang, Dengke Yang

**Affiliations:** ^1^ University of Science and Technology Beijing No. 30 Xueyuan Road, Haidian District Beijing 100083 China; ^2^ Peking University No. 5 Yiheyuan Road Haidian District Beijing 100871 P. R. China; ^3^ Department of Ophthalmology Beijing Anzhen Hospital Capital Medical University Beijing 100029 P. R. China; ^4^ Kent State University 1425 Lefton Esplanade Kent OH 44242 USA

**Keywords:** liquid crystal elastomer, structural color change, mechanochromic liquid crystalline elastomer

## Abstract

The ability of some animals to rapidly change their colors can greatly improve their chances of escaping predators or hunting prey. A classic example is cephalopods, which can rapidly shift through a wide range of colors. This ability is based on the synergetic effect of the change of pigmentary and structural colors exhibited by their own two categories of color‐changing cells: supernatant chromatophores offer various pigmentary colors and lower iridophores or leucophores reflect the different structural colors by adjusting their periodicities. Here, a mechanochromic liquid crystalline elastomer with force‐induced synergetic pigmentary and structural color change, whose mechanosensitivity is enhanced by the stress‐concentration induced by the doped nanoparticle, is presented. The materials have a large color‐changing gamut and high mechanochromic sensitivity, which exhibit great potential in the field of mechanical detectors, sensors, and anti‐counterfeiting materials.

## Graphical abstract

A mechanochromic liquid crystalline elastomer is presented with force‐induced synergetic pigmentary and structural color change, whose mechanosensitivity is enhanced by the stress‐concentration induced by the doped nanoparticle. The materials have a large color‐changing gamut and high mechanochromic sensitivity, which exhibit great potential in the field of mechanical detectors, sensors, and anti‐counterfeiting materials.

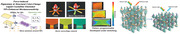

## 1 Introduction

In nature, some lives have developed various special abilities in a long evolutionary process of survival, camouflage is one of the effective means to increase their chances of escaping predators or hunting prey.^[^ ^1^ ^]^ Cephalopods, like the octopus and squid, can quickly change their color in a wide color range, because they have one of the best color‐changing mechanisms of all these species.^[^ ^2^ ^]^ Color‐changing cells are found in two categories in cephalopods: small pigmented organs (chromatophores) and structural reflector cells (iridophores and leucophores).^[^ ^3^ ^]^ When cephalopods experienced stimulation from the environment, their muscles govern the stretching or squeezing of chromatophores to quickly alter the hue of their body pigment cells as a response. Their structural colors can also be varied by thickening or thinning the protein layer that contains structural reflector cells. They can exhibit a diverse range of optical effects through the manipulation of synergetic pigmentary and structural color change.^[^ ^4^, ^5^ ^]^ Therefore, it is doubtless that a bionic mechanochromic material, with mechanochromism mechanisms like the cephalopod, can achieve richer optical properties and carry more information.^[^ ^6^, ^7^ ^]^

Recently, some biomimetic mechanochromic materials based on cholesteric liquid crystals (CLCs) have been reported.^[^ ^8^, ^9^, ^10^, ^11^ ^]^ Kizhakidathazhath et al. reported a mechanochromic cholesteric liquid crystals elastomer (CLCE) film using two‐stage thiol–acrylate Michael addition and photopolymerization reaction that resulted in a robust, rapid, and reversible mechanochromic response to CLCE film stretching.^[^ ^12^ ^]^ Ma et al. and Hussain et al. designed the elastomers which have obtained the mechanochromic change in the structural color of the materials by preparing CLCE films, respectively.^[^ ^13^, ^14^, ^15^ ^]^ However, the structural color of CLCE has some disadvantages not to be ignored, including a narrow color‐changing gamut range, low contrast ratio and brightness, and the inevitable angle dependence.^[^ ^16^ ^]^

Spiropyran‐based molecules (SPBMs) are a type of classical mechanochromic molecules that have received a lot of attention.^[^ ^17^, ^18^, ^19^ ^]^ SPBMs is a multistimulus response molecules that can switch from a spiro (SP) structure with no color to a merocyanine (MC) structure with color in response to environmental stimuli like heat, pH, light, and force.^[^ ^20^, ^21^ ^]^ According to Davis et al., mechanophore‐linked linear poly(methyl methacrylate) (PMMA) with a high elastic modulus was produced using biofunctionalized spiropyran as the mechanophore.^[^ ^22^ ^]^ The active core in mechanochromic pigment molecules can be mechanically triggered, as a result, the conjugation or charge state of the pigment molecules will be activated to absorb a specific wavelength of light.^[^ ^23^ ^]^ Other polymers with the property of force‐induced pigmentary color change are also obtained in the systems with high elastic modulus or very high elongation, such as PU,^[^ ^24^, ^25^ ^]^ hydrogels,^[^ ^26^ ^]^ and so on. In these systems, the sample usually needs to be stretched to a high elongation for triggering the functional core. However, the polymer systems are non‐elastic, it can't restore to the initial state after external force removal. Therefore, the reversibility and fatigue of spiropyran mechanochromic polymers are very challenging.

Introducing the SPBMs into a CLCE system can combine the force‐induced pigmentary and structural color‐changing, and the obtained materials can exhibit a wider color gamut and higher contrast and brightness. Furthermore, the spiropyran mechanochromic liquid crystal elastomer (LCE) can completely restore to the initial state after external force removal, which can solve the reversibility and fatigue of the material. However, the mechanical strength of the reported CLCEs, which mostly were fabricated from some polymerizable acrylate‐based monomers, cannot satisfy the required enough stress strength to trigger the mechanochromic core.^[^ ^27^ ^]^ Moreover, the reported mechanochromic polymers based on SPBM usually demand a high elongation to trigger the functional core.^[^ ^24^ ^]^ Nevertheless, many applications of mechanochromism materials, such as sensors, require enough high stress sensitivity to measure the level of the force value in real‐time. Therefore, it is essential to enhance the mechanosensitivity of the mechanochromic system. Introducing nanoparticles (NPs) into the mechanochromic system to obtain composites with heterogeneous interfaces, in which stress concentrates at the interface between mechanically dissimilar materials, should be an effective approach.^[^ ^28^, ^29^ ^]^

In this work, a bionic liquid crystal elastomer (BLCE) film that imitates the color‐changing mechanism of cephalopods is reported. A cross‐linkable SPBM which can appear pigmentary color, fluorescence, and mesomorphism after being triggered has been reported in our previous work.^[^ ^30^ ^]^ This SPBM is used as a functional core of the force‐induced pigmentary color‐changing unit; a CLCE with tunable helical pitches is used as the force‐induced structural color‐changing unit. Two units are combined into a compound system, modified NPs are doped to improve mechanochromic sensitivity. The obtained BLCE film has a large color‐changing gamut and high mechanochromic sensitivity by combing two mechanochromism mechanisms like cephalopods. These kinds of bionic mechanochromic materials have tremendous development potential in a multitude of areas, such as smart skin, smart displays, sensors, and camouflage materials.

## 2 Result and Discussion

**Figure 1:**
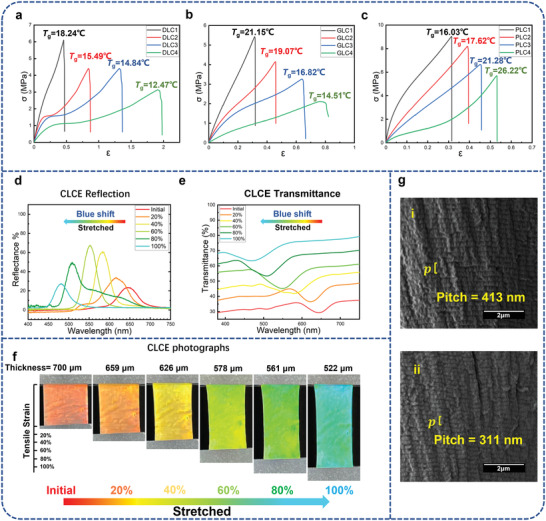
Characterization data of CLCE. The stress–strain curve and the *T*_g_ of samples a) DLC1‐4, b) GLC1‐4, and c) PLC1‐4, respectively. d) Reflection spectra, e) transmittance spectra, and f) photographs of the CLCE Sample CLC1 at different elongations. g) Cross‐section SEM images of Sample CLC1 at (i) 0% and (ii) 100% elongation, respectively.

As an elastomer material, excellent mechanical properties are very important. Desired BLCE films should have enough elongation at break to exhibit a force‐induced large structural color change gamut. Moreover, a high elastic modulus is demanded to ensure that enough high stretching stress generates to activate the pigmentary mechanophore during the process of stretching. To obtain an LCE film with desired mechanical properties, the material composition and preparation condition of the film were systematically investigated. The matrix of the LCEs was prepared from liquid crystalline polymeric monomer RM82 and a kind of dithiol. Three kinds of different dithiols EDDET, EGBTG, and BPAT were used to react with RM82, their chemical structures are shown in Figure S1 (Supporting Information). The samples named DLC1‐4, GLC1‐4, and PLC1‐4 were prepared from EDDET, EGBTG, and BPAT, respectively, and their material compositions can be seen in Table S1 (Supporting Information). All the samples were prepared into rectangles with 700 µm thickness and cut into dumbbell shapes. The stress–strain curves of Samples DLC1‐4, GLC1‐4, and PLC1‐4 are shown in **Figure** **1**a–c, respectively. It was found that as the disulfide content increased, the elasticity modulus and glass transition temperature (*T*_g_) of the samples decreased, whereas the breaking elongation increased. It can be attributed to the decrease of crosslinking density with the disulfide content increasing within the matrix. Samples PLC1‐4 showed higher elasticity modulus, but they and samples GLC1‐4 all have very low breaking elongations, which cannot satisfy enough deformation demand to cover a wide range of mechanochromism. In a contrast, Sample DLC3 has an appropriate elasticity modulus and 137% of large breaking elongation, which was used as the matrix material of CLC's in the following studies.

Cholestic is a unique liquid‐crystalline phase with a periodic helical structure, exhibiting special selective Bragg reflection to light.^[^ ^31^, ^32^ ^]^ And this makes CLCs exhibit visible specific polarized structural color when the selective reflection band (SRB) centers in the wavelength range of visible light.^[^ ^33^, ^34^ ^]^ The reflection structural color wavelength (*λ*), according to Bragg's law, can be given by the following equation

(1) λ=n·p·cosθ

where *n* is the refractive index of liquid crystals, and *p* is the helix pitch of the matrix.^[^ ^35^ ^]^ It can be known that the reflection wavelength *λ* can be adjusted by changing the *p* of the CLCs from the equation. Therefore, CLCE materials can exhibit extremely high mechanochromic sensitivity during deformation due to their *p* being altered.

To endow the LCE with a structural color, a chiral polymerizable monomer named RIA that was synthesized in our lab was introduced into the above‐selected system.^[^ ^36^ ^]^ A cholesteric LCE named Sample CLC1 was prepared. As a means of showing the mechanochromic performance of CLCE films, Sample CLC1 was prepared into rectangular pieces with a thickness of 700 µm and a width and length of 10 mm. Reflection spectra, transmittance spectra, real photographs, and polarized optical microscopy (POM) images in reflection mode of the sample at the different uniaxial stretched elongations are shown in Figure 1d–f and Figure S2 (Supporting Information), respectively. When the elongation of the film increases from 0 to 100% of its initial length, the reflection wavelength peak of Sample CLC1 blueshift from 648 to 485 nm, and the corresponding transmittance spectra verify the blueshift behavior during this process. Meanwhile, the thickness of the film decreases from 700 to 522 µm with stretching, and the structural color of the sample changed from red‐orange to cyan continuously (Figure 1f and Video S1, Supporting Information). Furthermore, the scanning electron microscopy (SEM) image of the cross section of Sample CLC1 shows that the pitch of the CLC is 413 nm in the initial state (Figure 1g‐i) and 311 nm after uniaxial stretching to 200% of the original length (Figure 1g‐ii), indicating that the pitch and the reflection structural color wavelength are basically in agreement with Bragg's law. Accordingly, the blueshift of the structural color wavelength of the CLCE film is due to a continuous decrease in the helix pitch of CLCs induced by stretching.

As expected, the structural color of pure CLCE exhibits a rapid discoloration response to the mechanical stimulation, but its contrast ratio and color‐change gamut are deficient. Imitating the structural and pigmentary dual mechanochromism of cephalopods will be beneficial for remedying the above defect. To avoid the interference from structural color and clearly present the pigmentary mechanochromism, at this stage, a SPBM as the pigment mechanophore was doped into the DLC3 system without the chiral monomer to study the pigmentary mechanochromism. The SPBMs were prepared based on our previous work, whose mechanochromism behavior comes from force‐induced ring‐opening reactions under stretching.^[^ ^26^ ^]^ After the C—O bond was ruptured by the force, an isomeric form called MC generated and absorbed the light of about 570 nm to present a violet color.^[^ ^37^ ^]^

To investigate the effect of pigmentary mechanophore content on the pigmentary mechanochromic, different contents of SPBMs were doped into the DLC3 system to prepare the LCE Samples SP0‐4. The contents of SPBMs were 0, 1, 2, 3, and 4 wt% in Samples SP0‐4, respectively. As illustrated in Figure S4 (Supporting Information), the MC form of the SPBMs exhibits a characteristic absorption peak at 570 nm. Therefore, the absorption peak at 570 nm will occur when the ring‐opening isomerism is triggered by stretching.

**Figure 2:**
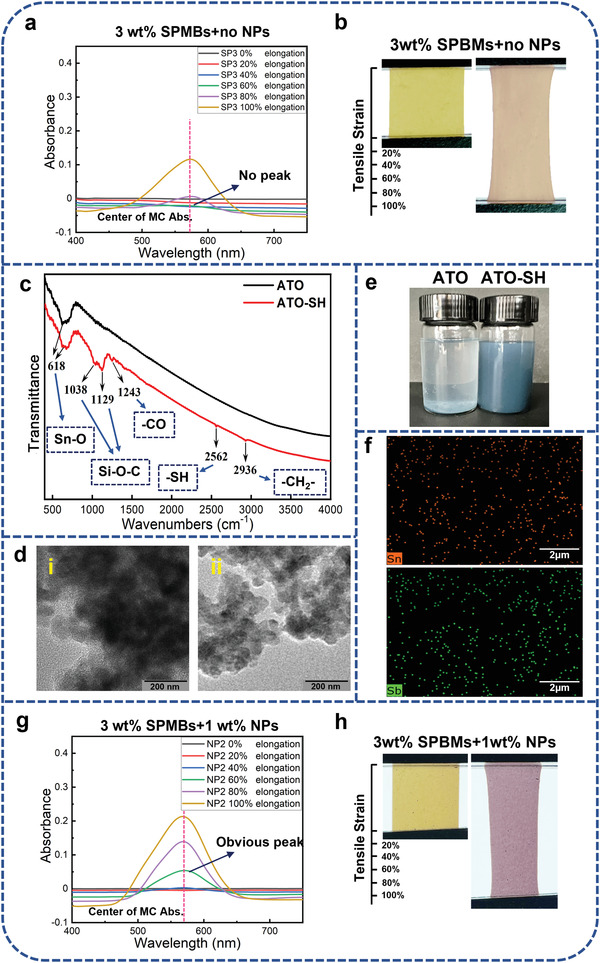
a) Absorption spectrums of Sample SP3 at different elongations. b) Photograph of Sample NP2 at 0% and 100% elongation. c) FTIR spectra of ATO nanoparticles before and after modification by KH590. d) TEM images of (i) the virgin ATO NPs and (ii) the modified ATO NPs whose dispersibility is obviously improved. e) Photos of the virgin and the modified ATO NPs dispersing in the organic solvent (tetrahydrofuran). f) SEM‐EDS mapping diagram of Sample NP2, the elements of Sb and Sn are evenly distributed in the matrix. g) Absorption spectra of Sample NP2 at different elongations. h) Photograph of Sample NP2 at 0% and 100% elongation.

All the samples were prepared into rectangle films of 700 µm thickness and 10 mm width. The samples were uniaxially stretched along with the orientation of the LCs in the films with a 10 mm initial length, and the absorption spectra were measured during the stretching processes as shown in **Figure** **2**a and Figure S5 (Supporting Information). As the content of the SPBMs is less than 2%, discoloration is hardly detected throughout the whole stretching process. The absorption peaks have been observed at about 570 nm in the stretched length which is greater than 60% of the initial length for Samples SP2‐4; however, there is no significant absorption peak for the elongation which is less than 40%.

From the absorption spectra and the real color variations of all the samples during the stretching process, distinct mechanochromic behaviors can be observed when the content of the SPBMs exceeds 3 wt%. However, too many SPBMs would make the sample show a deep initial color, the content of the SPBMs was fixed at 3 wt% in the following study. The absorption spectrum and the real photos of Sample SP3 with 3 wt% SPBMs during the stretching process are shown in Figure 2a,b, an absorption peak at about 570 nm occurs and increases with stretching while the color of the film changes to pale violet from pale yellow.

Therefore, the mechanochromic behavior was preliminarily achieved by introducing the SPBMs into the LCE system. However, the light absorption can only be detected until the length of the film of SP2‐4 were stretched more than 60% elongation, while the discoloration is too weak to be perceived by the naked eye until the samples were stretched up to 80% elongation. The low mechanosensitivity of this system is not satisfactory, the pigmentary color change is not obvious when the tensile strain is less than 100%. To enhance the mechanosensitivity, we attempt to introduce NPs into the above system to increase the mechanotransduction efficiency. Antimony‐doped tin oxide (ATO) nanoparticles (NPs) have a high transmittance rate of visible light and good thermostability, which were ideal doped particles for the color sensitive film.

However, virgin ATO NPs have poor intermiscibility in an organic material system and bad dispersibility induced by agglomeration. To enhance the dispersibility and interaction of ATO NPs in the system, the ATO NPs were modified by 3‐mercaptopropyltriethoxysilane (KH590). The FTIR spectra of ATO and KH590 modified ATO are shown in Figure 2c, which illustrates the vibration absorption peak of Sn—O at ≈618 cm^−1^. The vibration absorption peak of Si—O—C appears near 1038 and 1129 cm^−1^ after nano‐ATO particles are surface‐modified by KH590. The new absorption peaks have been observed at 2936 and 2562 cm^−1^, which correspond to the C—H stretching vibration peak and the S—H stretching vibration peak of the methylene group in KH590, respectively. The ATO NPs achieved good dispersion after being modified by KH590. Comparatively to the virgin ATO NPs, the modified ATO NPs have a brighter TEM image, as shown in Figure 2c, which clearly illustrates the dispersed particles with a diameter of ≈70 nm. The improved dispersibility of modified ATO NPs in the organic solvent (tetrahydrofuran) is demonstrated in Figure 2e.

Then, the modified ATO NPs were introduced into the material system of Samples SP3 to prepare Sample NP1, NP2, and NP3, in which the content of the modified ATO NPs was 0.5, 1.0, and 1.5 wt%, respectively. All the samples were prepared into rectangle films of 700 µm thickness and 10 mm width. The SEM‐EDS mapping diagram of Sample NP2 is shown in Figure 2f, the elements of Sn and Sb are evenly dispersed in the matrix, implying that the ATO NPs are evenly distributed in the polymer matrix. Figure 2g shows the absorption spectrums of Samples NP2 with different stretched length ratios, and the absorption spectrums of Sample NP1 and NP3 are shown in Figure S6 (Supporting Information). The absorption peak from the MC form of the SPBMs is observed in all samples when the stretched length is over the critical value. To compare Samples NP2 and SP3, NP2 has the more intense absorption peak of MC form when the stretched length is up to 40% elongation, which means it obtains a lower critical stretched length of discoloration after introducing modified ATO NPs. The photograph of Sample NP2 at 0% and 100% elongation are shown in Figure 2h, the intensity of the force‐induced pigmentary color change is far stronger than the SP3 system which does not contain the ATO NPs.

**Figure 3:**
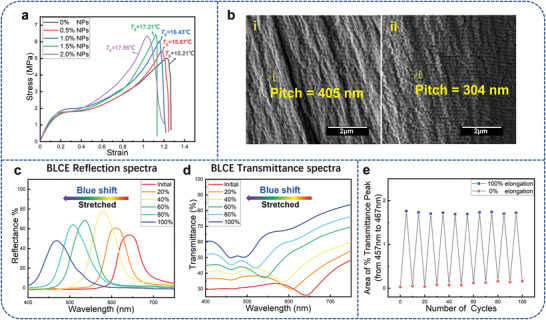
a) Stress–strain curve and the *T*_g_ of the samples with different NPs contents. b) Cross‐section SEM image of Sample BLC1 at the initial state (i) and the state with a 100% elongation (ii), corresponding to the different pitches of 405 and 304 nm, respectively. c) Reflection spectra and d) transmittance spectra of the BLCE sample at different stretching length elongation, where the wavelength is blueshift as it is stretched, and the wavelength range is wider than that of CLCE under the same stretching conditions. e) Reversibility of the mechanically responsive pigment color of the BLCE film under 100% elongation.

The tensile stress–strain curve and the *T_g_* of the samples with different NPs content are shown in **Figure** **3**a, the elasticity modulus and the *T*_g_ rise continually with the NPs increasing. Obviously, the modified ATO NPs with multi‐thiol functional groups increased the crosslinking density as crosslinking centers. Therefore, the mechanosensitivity of the system is significantly enhanced, which is due to an increased crosslinking density and a stress concentration effect introduced by the NPs.^[^ ^38^, ^39^, ^40^, ^41^, ^42^ ^]^

By introducing NPs and SPBMs into the CLCE polymer matrix, a BLCE sample (BLC1) is obtained according to the material composition in Table S3 (Supporting Information). The SEM images of a cross section of the initial state and a stretched state with a 100% elongation of Sample BLC1 are shown in Figure 3b‐i,ii, respectively. Sample BLC1 has a helical pitch of ≈405 nm at the initial state, which corresponds to the double value of the distance between the two stripes in the SEM image; the helical pitch turns to be ≈304 nm when the elongation is 100%. Compared with the CLCE sample, the pitch values and the variation tendency are similar before and after stretching. According to Bragg's law, the BLCE sample should have the same structural color performance as the CLCE sample.

However, the reflection peaks of Sample BLC1 blueshift from 646 to 465 nm with the elongations increasing (Figure 3c). Compared with Sample CLC1 and the previously reported CLCE films,^[^ ^13^, ^43^ ^]^ Sample BLC1 has a broader shifting range of the reflection peak wavelength and a relatively higher reflectivity. It is noteworthy that the wavelength peak shift of Sample BLC1 has a sharper movement when the elongation changes from 60% to 80%. The transmittance spectra of the BLCE sample (Figure 3d) show that a valley at about 462 nm emerges and grows with the elongation increasing, which can attribute to the absorption of the MC form of the SPBMs. It can be seen that the reflectance of the CLCE film decreased markedly at the high elongation due to the film thickness decreases from Figure 1d. For example, at 100% elongation, the reflectance of CLCE film is less than 30%, however, the reflectance of BLCE film is ≈50% at the same condition. Therefore, the performance of the BLCE is obviously better than the classical structural color film.

According to our previous work, once the pigmentary mechanophore SPBMs convert to the MC form, a characteristic red fluorescence is triggered under the 365 nm UV light. The characteristic red fluorescence can be used to confirm the occurrence of the force‐induced stimuli‐responsive behavior of the SPBMs. Under a 0.05 mW cm^−2^ 365 nm UV light, the fluorescence was barely detected at the initial state; the fluorescence was still faint at the state with the elongation of 0%–60%; the red fluorescence turned to be more and more intensive with stretching when the elongation exceeded 60% (Figure S7a and Video S2, Supporting Information). As a comparison, no fluorescence was observed in a CLCE sample which was also stretched at the same condition (Figure S7b and Video S3, Supporting Information).

Therefore, the force‐induced pigmentary color from SPBMs was observably triggered at a high elongation. The POM images in reflection mode of the BLCE film at the different uniaxial stretched elongations are shown in Figure S8 (Supporting Information), which exhibit the typical planar textures of the cholesteric liquid crystal with the blueshifting color with increasing elongation. Furthermore, the initial structural color of Sample BLC1 is red‐orange, which accord with the wavelength of the reflection peak. However, the color of BLCE turns to blue at the state with the 80% elongation, the reflection wavelength is distinctly lower than the value calculated by Bragg's law with the actual pitch from the SEM image. Therefore, the presented color of the BLCE film is the combined pigmentary and structural color at a state with high elongation. Compare with Sample CLC1, the reflection peak wavelength of BLC1 has a larger blueshift at the same elongation. The enhanced mechanosensitivity and the wider color‐changing range come from the combined effect of the mechanochromic behavior of the structural color and the pigmentary color. Figure 3e and Figure S9 (Supporting Information) show the high cycle stability of the BLCE film after a continuous stretching and releasing test. Therefore, Sample BLC1 exhibits superior mechanochromic performances than Sample CLC1.

**Figure 4:**
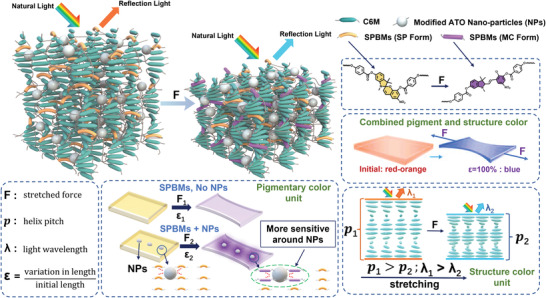
Mechanochromic mechanisms schematic diagram of the BLCE film. The composite system is composed of a structural color unit and a pigmentary color unit with high mechanosensitivity enhanced by NPs.

The schematic diagram of the BLCE mechanochromic mechanism is shown in **Figure** **4**, and this system combines the structural and pigmentary mechanochromism. When the BLCE film is uniaxially stretched from the initial state (the elongation is less than 60%), the color change from the SPBMs is very slight due to the critical elongation for the pigmentary mechanophore is not reached. Therefore, the mechanochromism behavior of the BLCE films is dominated by the structural color changes from the variation of the helical pitch at a state with low elongation. When the elongation of the BLCE films exceeds 60%, the pigmentary mechanochromism is triggered because some SPBMs in the matrix is converted to the MC form which exhibits an intensive pigmentary color. And the doped ATO NPs modified with the mercapto group increase the crosslinking density and offer a stress concentration effect induced by the interfacial effect between the polymer matrix and the spherical NPs. These combined effects markedly enhance the mechanosensitivity of the color of the system.

**Figure 5:**
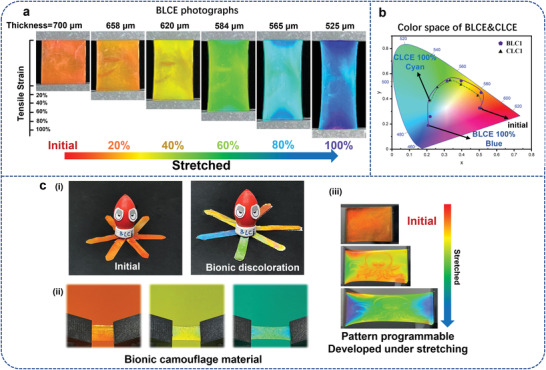
a) Photograph of the BLCE sample at different elongations, in which color continually changes from red‐orange to blue. b) CIE 1931 color space diagram of a comparison between the gamut of the samples of BLCE and CLCE under the same stretching conditions. c) Dynamic mechanoresponsive behavior of BLCE films. d) Picture of cephalopods camouflaged in the environment in nature (i), picture of the cephalopod doll with BLCE as the tentacles in the initial state (ii), and being stretched to different colors (iii), and the photographs of the BLCE film with a dynamic octopus pattern at different elongations.

As shown in **Figure** **5**a and Video S4 (Supporting Information), the color of Sample BLC1 can change from red‐orange to blue as the elongation goes from 0% to 100%. In addition, the wider mechanochromic range of Sample BLC1 is demonstrated in the average color coordinates based on the CIE 1931 color space diagram (Figure 5b) than Sample CLC1. Furthermore, the mechano‐optical properties of BLCE were investigated.

Cephalopod's color can relax or tense the chromatophores in their skin to adjust their body color, which allows them to camouflage into their surroundings. Similarly, the BLCE film can easily and rapidly change its color in a large range, which benefits from the force‐induced synergetic pigmentary and structural color‐change mechanisms (Figure 5d‐i). And the wider color‐changing range endows the film with the ability to merge into the various complex color backgrounds (Figure 5d‐ii). In addition, as shown in Figure 5d‐iii and Video S5 (Supporting Information), an anti‐counterfeiting model is demonstrated. An octopus pattern is hidden in the film, which cannot be detected at the initial state. The pattern will emerge and change color with stretching. If some tailored signal is used as the hidden pattern, a dynamic anti‐counterfeiting mode can be achieved.

## 3 Conclusion

In conclusion, a mechanochromic bionic liquid crystalline elastomer with force‐induced synergetic pigmentary and structural color change, whose mechanosensitivity was enhanced by the stress‐concentration induced by the doped functional nanoparticle. The BLCE film combines the SPBMs as a mechanophore for the pigmentary color change, the unique helix structure of the CLCE for the structural color change, and doped NPs to improve mechanochromic sensitivity. It has a large color‐changing gamut and high mechanochromic sensitivity by combing two mechanochromeism mechanisms like cephalopods. Due to the wide color‐changing range and the high mechanosensitivity, the BLCE film has great potential in the field of mechanical detectors, sensors, and anti‐counterfeiting materials.

## 4 Experimental Section

### Materials

The liquid crystalline polymeric monomer RM82 was purchased from Jiangsu Hecheng Display Technology Co., Ltd.; the dithiols EDDET, EGBTG, and KH590 were purchased from Bide Pharmatech Ltd. The photoinitiators [bis(2,6‐difluoro‐3‐(1‐hydropyrro‐1‐yl)‐phenyl)titanocene] and amine catalysts (butylamine) were purchased from Energy Chemical Co., Ltd. The ATO NPs were purchased from Aladdin Bio‐Chem Technology Co., Ltd.

### Characterizations

The photopolymerization of all polymer samples was used as an ultraviolet (UV) light (520 nm) source with a LED lamp (FUV‐6BK, Bangwo Elec. Technologies Co., Ltd.). And the tensile tests of the samples were done using a universal tensile meter (CMT6103, MTS Systems (China) Co., Ltd., Shenzhen, China) with the standard method of GB/T 1040.2‐2006. Differential scanning calorimetry (DSC) was performed using Mettler‐Toledo DSC3 operated at a scanning rate of 10 °C min^−1^ under a nitrogen atmosphere. The microscopic observations were taken by POM (Axio Scope A1 pol, Zeiss). The images of the morphology of nanoparticles were taken by a field emission electron microscope (JEM‐2100F JEOL Ltd.). The fracture of the CLCE and BLCE films and the EDS‐mapping images were observed by SEM (HITACHI S‐4800). Before observation, the fractured surface of the sample was coated by a 10 nm gold layer. The FTIR spectra were measured using FTIR Spectrometer (Perkin Elmer Spectrum Two). The absorption spectrum and the transmittance spectrum were taken by a Perkin Elmer Lambda 950 spectrophotometer. The absorption spectrum was set to zero by referencing samples which were unstretched. The reflection spectrum and the dynamic mechanoresponsive behavior were measured using a fiber optic spectrometer (Avaspec‐ULS2048, 360–1100 nm, white background) with a stabilized halogen light source (AvaLight‐HAL‐S‐Mini), and the dynamic mechanoresponsive behavior was under TimeSeries mode (function type was peak time). The central thickness of the samples at different stretched lengths was taken by thickness gauge (G6C Peacock Ozaki Mfg. Co., Ltd.).The fluorescent properties of samples were tested by fluorescent spectrometer (PerkinElmer, LS55).

### Methods

The ATO NPs were modified by KH590 before used. A 2 g of ATO NPs was mixed into an 80 mL mixed solution of water and ethanol at a ratio of 2:3, and a corresponding amount of KH 590 was mixed into the solution and refluxed for 12 h. The solids were centrifuged out of the solution, washed five times with water, and dried to obtain the modified ATO NPs.

The BLCE film was prepared by a thiol–acrylate Michael addition followed by photopolymerization. And the chemical structures of the RM82 and the chiral monomers RIA are shown in Figure S1a (Supporting Information). RM82 was used as LC monomers, it was mixed with SPBMs, NPs, photoinitiators, amine catalysts, chiral monomers, and disulfide. Afterward, the mixture was stirred at 50 °C for 20 min and dispersed in ultrasonic for 20 min, followed by casting between two glass substrates on a heat plate of 50 °C for 24 h to allow Michael addition, then irradiating the film with the light of a wavelength of 520 nm for 30 min to induce photoinitiated crosslinking. The BLCE film was obtained by peeling it away from glass substrates.

## Conflict of Interest

The authors declare no conflict of interest.

## Supporting information

Supporting Information

Supplemental Video 1

Supplemental Video 2

Supplemental Video 3

Supplemental Video 4

Supplemental Video 5

## Data Availability

Research data are not shared.
