# Supplementary material for: Force‐Induced Synergetic Pigmentary and Structural Color Change of Liquid Crystalline Elastomer with Nanoparticle‐Enhanced Mechanosensitivity
Source: Adv Sci (Weinh). 2022 Oct 30;9(36):2205325. doi: 10.1002/advs.202205325 (PMC9798961; doi:10.1002/advs.202205325)
Supplement: Supplementary file 1 — Supporting Information [file ADVS-9-2205325-s004.pdf]

ADVANCED SCIENCE
Open Access

Supporting Information

for *Adv. Sci.*, DOI 10.1002/advs.202205325

Force-Induced Synergetic Pigmentary and Structural Color Change of Liquid Crystalline Elastomer with Nanoparticle-Enhanced Mechanosensitivity

Chang Sun, Shuoning Zhang, YunXiao Ren, Jianying Zhang, Jiyuan Shen, Shengyu Qin, Wei Hu, Siquan Zhu*, Huai Yang* and Dengke Yang*

Supporting Information

Force-induced Synergetic Pigmentary and Structural Color-Change of Liquid Crystalline Elastomer with Nanoparticle-Enhanced Mechanosensitivity

Chang Sun, Shuoning Zhang, YunXiao Ren, Jianying Zhang, Jiyuan Shen, Shenyu Qin, Wei Hu, Siquan Zhu*, Huai Yang*, Dengke Yang*

1. Materials

The dithiols EDDET, and EGBTG were purchased from Bide Pharmatech Ltd. BPAT was synthesized following a previously described method[1].

Characterizations

To display the fluorescent properties of the samples, the samples were exposed under an ultraviolet (UV) light (365 nm) source with a LED lamp (FUV-6BK, Bangwo Elec. Technologies Co., Ltd.) with 10% of the maximum power. To observe the cross-section of CLCE and BLCE with different stretching rates, the samples were cut into two pieces of the same size which suitable for SEM observation, one piece was stuck on the base plate directedly, and another one was stretched to a certain stretching rate and stuck on the base plate with keeping the stretching state.

To measure the Dynamic mechanoresponsive behavior of BLCE films, the sample was set on the stretching machine and placed on the white background. The sample was measured by fiberoptic spectrometer under TimeSeries mode, and the applied strain was changed by the stretching machine during the measurement.

Method

The pattern-programmed BLCE was prepared using various degrees of polymerization to form different moduli area which developed patterns under stretch.

2. Supplementary Figures and Results

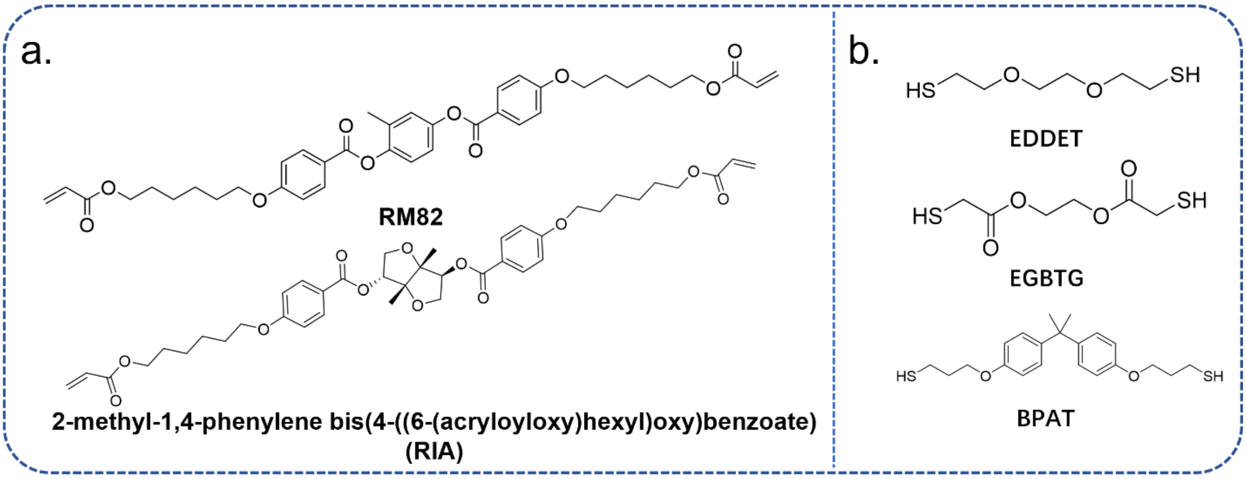

Figure S1. a) The chemical structures of RM82 and the chiral monomers RIA. b) The chemical structures of the disulfides (EDDET, EGBTG, and BPAT).

Table S1. Chemical formulations and monomers molar ratio for the samples.

Samples	*Disulfide*	*Molar ratio of disulfide to RM82*	*Mass of RM82 (mg)*	*Mass of disulfide (mg)*
DLC1	EDDET	6:10	300	48.8
DLC2	EDDET	7:10	300	57.0
DLC3	EDDET	8:10	300	65.1
DLC4	EDDET	9:10	300	73.2
GLC1	EGBTG	6:10	300	56.3
GLC2	EGBTG	7:10	300	65.7
GLC3	EGBTG	8:10	300	75.1
GLC4	EGBTG	9:10	300	84.5
PLC1	BPAT	6:10	300	100.9
PLC2	BPAT	7:10	300	117.7
PLC3	BPAT	8:10	300	134.5
PLC4	BPAT	9:10	300	151.3

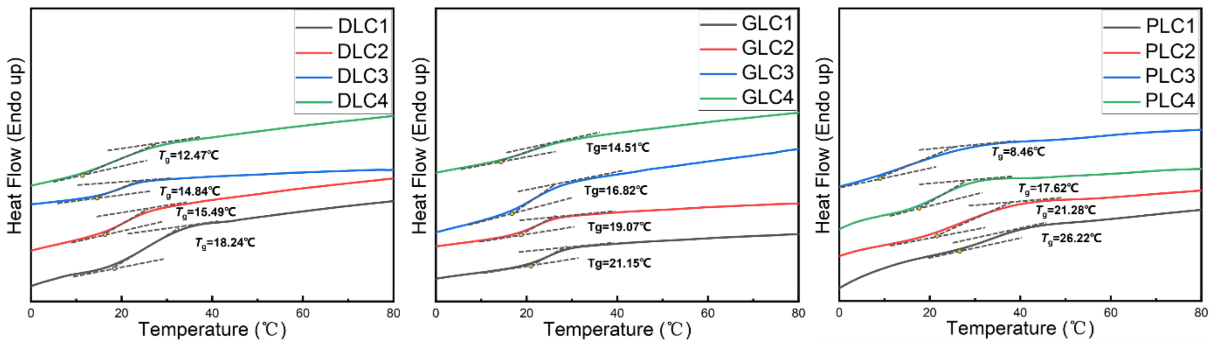

Figure S2. DSC curves of the samples DLC1-4, GLC1-4, and PLC1-4, respectively.

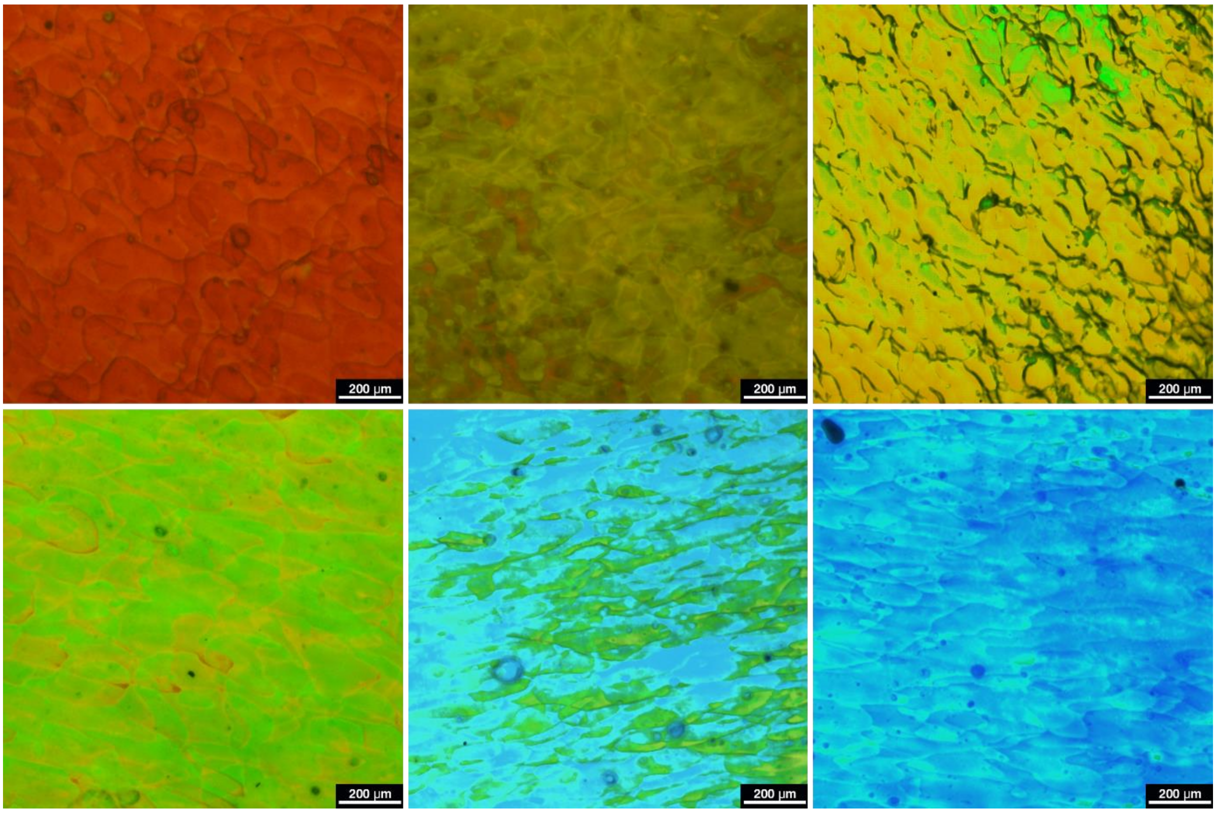

Figure S3. The POM images of the CLCE film in different stretched length ratio (from 0 to 100%).

Samples	*Mass of RM82 (mg)*	*Mass of EDDET (mg)*	*Mass of chiral monomers (mg)*	*Mass of SPBMs (mg)*	*Mass of NPs (mg)*
SP0	300	65.1	0	0	0
SP1	300	65.1	0	3.7	0
SP2	300	65.1	0	7.3	0

SP3	300	65.1	0	11.0	0
SP4	300	65.1	0	14.6	0
NP1	300	65.1	0	11.0	1.9
NP2	300	65.1	0	11.0	3.7
NP3	300	65.1	0	11.0	5.6

Table S2. Chemical formulations and monomers molar ratio for the samples.

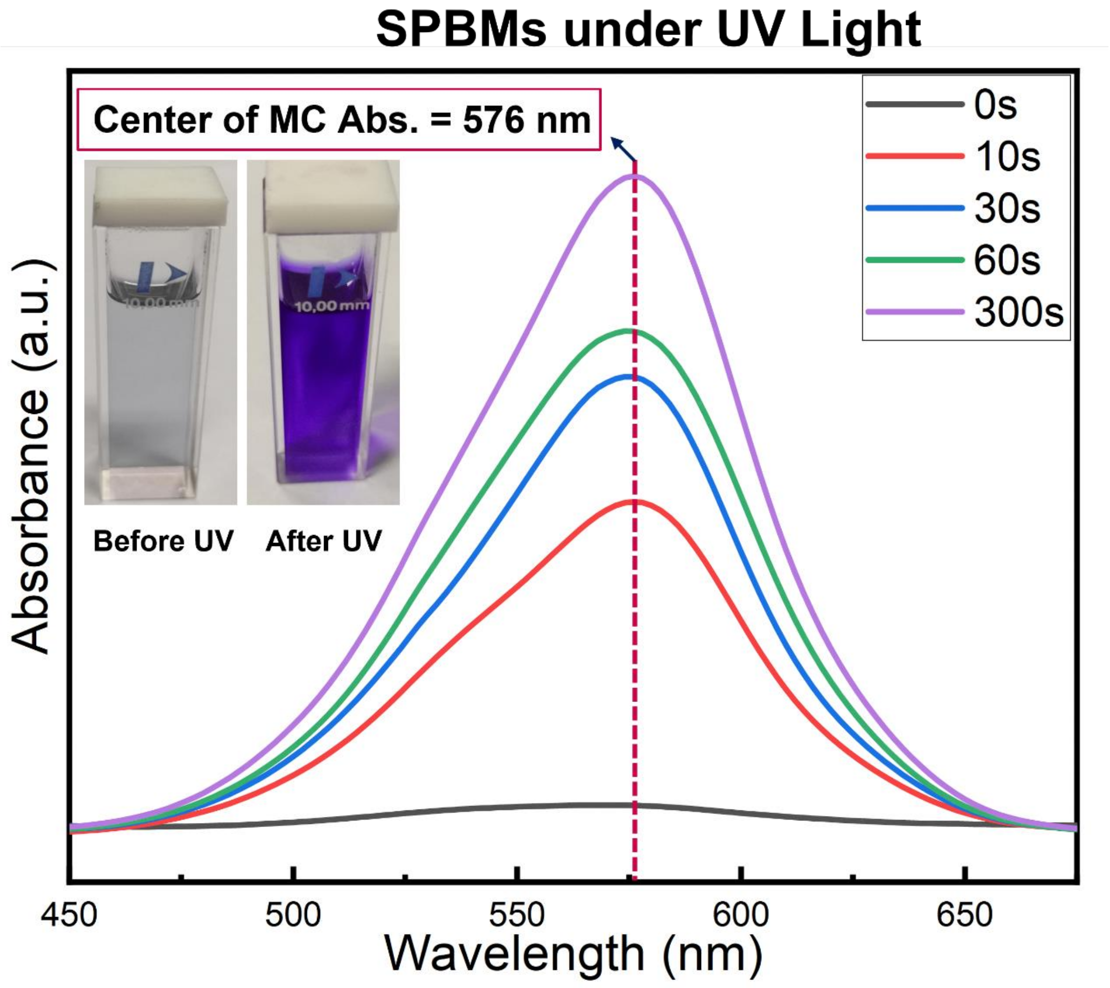

Figure S4. The absorption spectra of SPBMs when exposed to UV light (wavelength=365nm) for different periods of time. The absorption peak is located at 576nm.

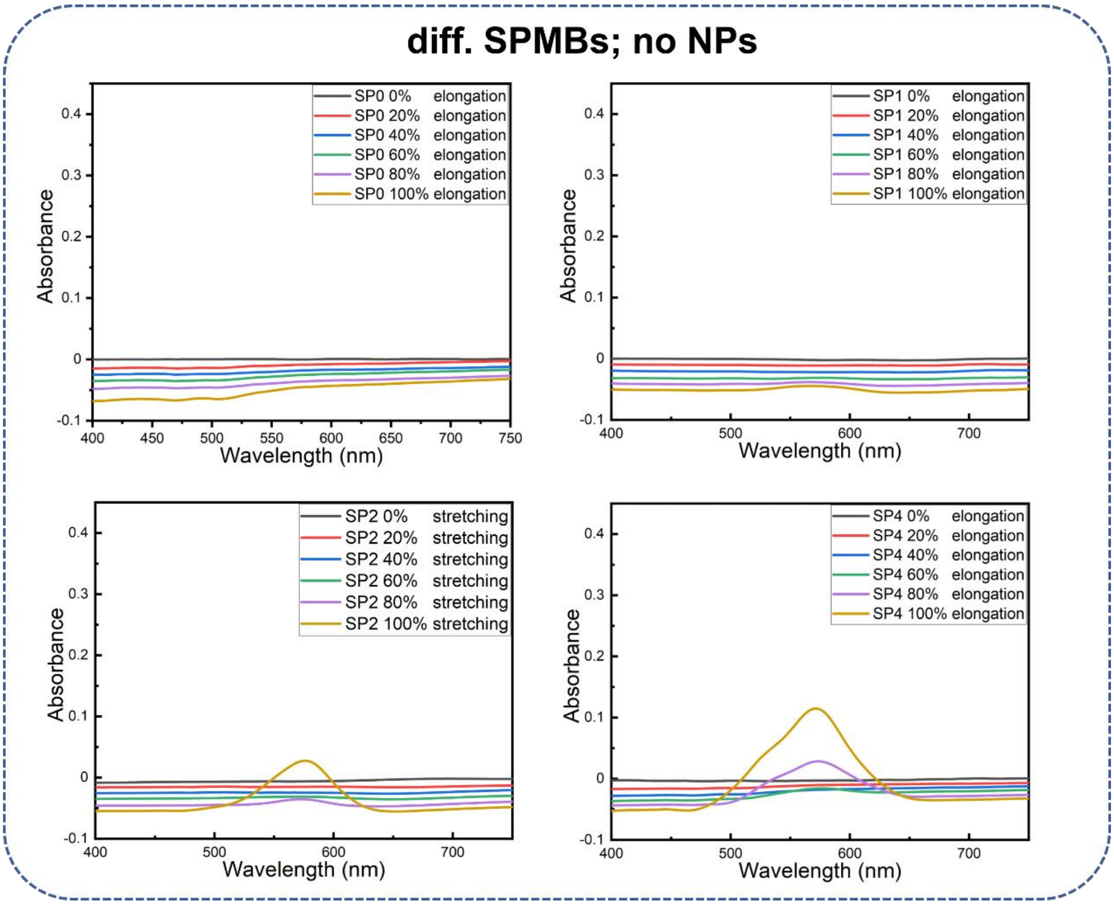

Figure S5. The absorption spectra of Samples SP0-2, 4 at different stretched length ratios, respectively.

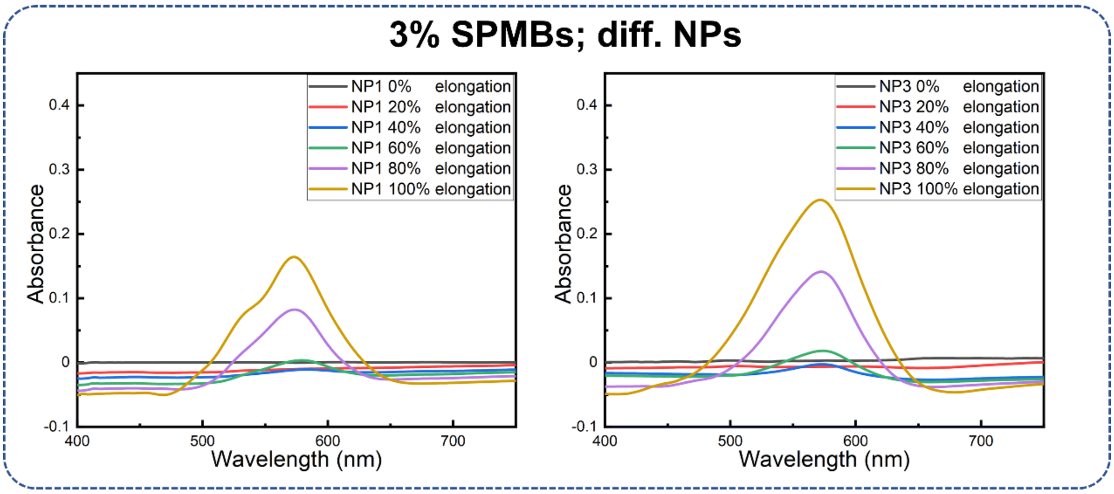

Figure S6. The absorption spectra of Samples NP1 and NP2 at different stretched length ratios, respectively.

Table S3.

Samples	*Mass of RM82 (mg)*	*Mass of EDDET (mg)*	*Mass of chiral monomers (mg)*	*Mass of SPBMs (mg)*	*Mass of NPs (mg)*
CLC1	300	65.1	25.6	0	0
BLC1	300	65.1	25.6	11.0	3.7

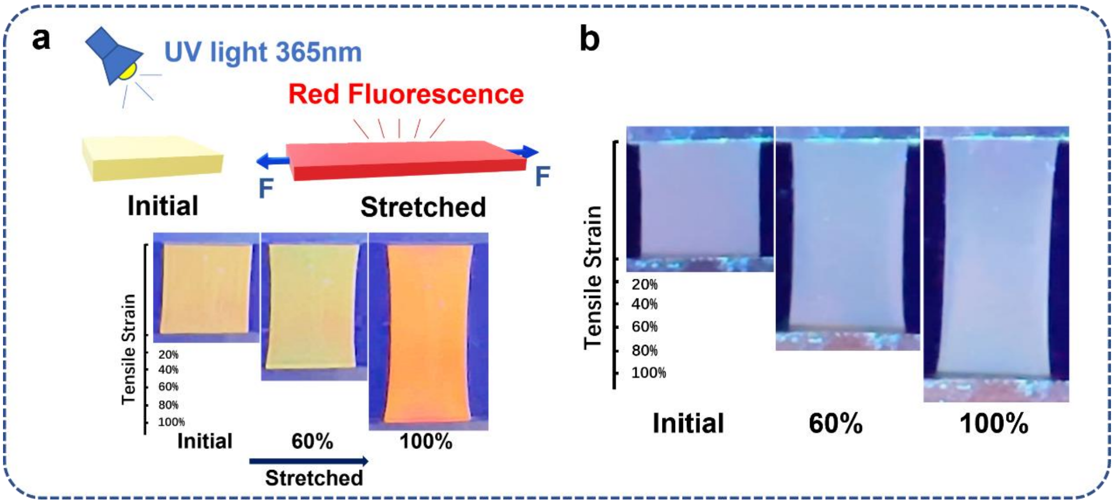

Figure S7. a) The schematic diagram of the BLCE film stretched under UV light and triggered the characteristic red fluorescence. And the photographs of the film at different stretching lengths under UV light show that the characteristic red fluorescence appears as the stretching length increases. b) The photographs of the CLCE film at different stretching lengths under UV light show that no fluorescence is triggered.

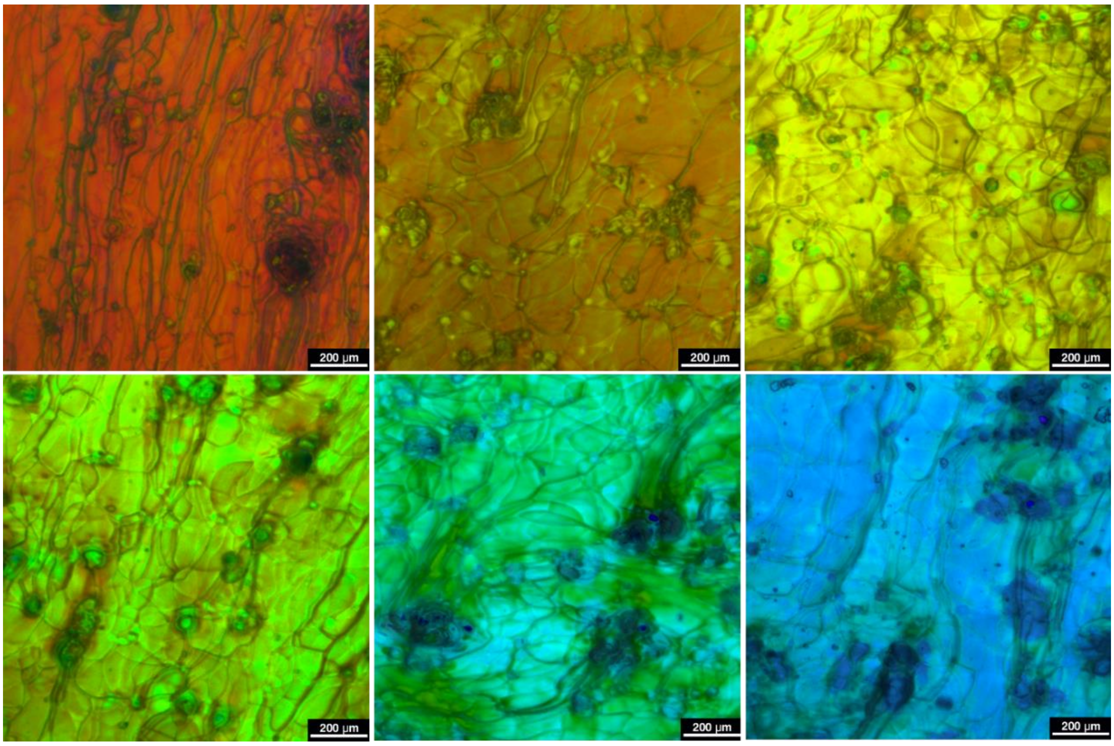

Figure S8. The POM images of the BLCE film at different stretched length ratios (from 0 to 100%)

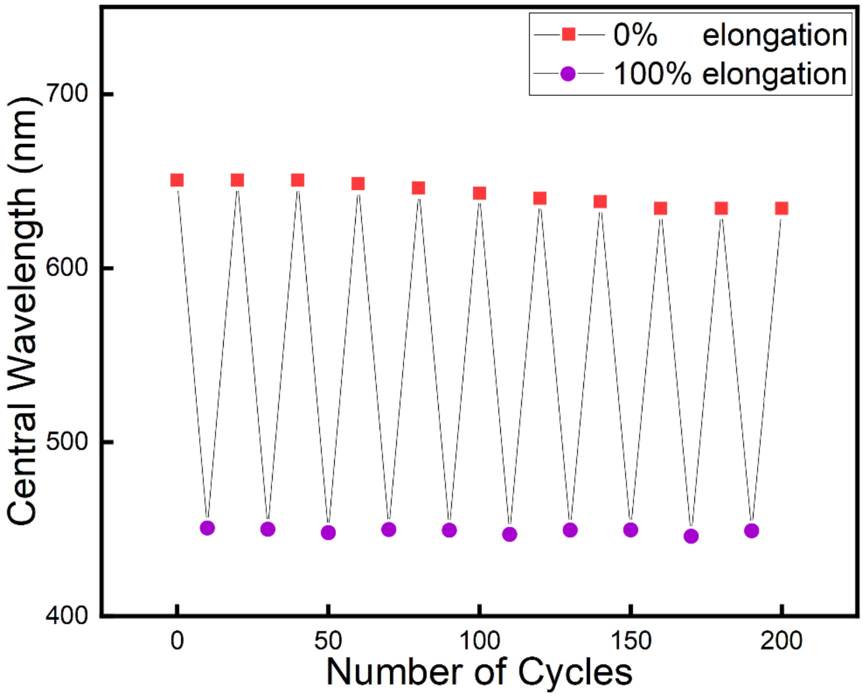

Figure S8. The reversibility of the mechanically responsive synergetic color of the BLCE film under 100% elongation

[1] W. Hu, M. Chen, L. Zhou, T. Zhong, X. Yuan, F. Chen, L. Zhang, *ACS Applied Materials & Interfaces* **2018**, 10, 22757.
